# Event detection and classification from multimodal time series with application to neural data

**DOI:** 10.1088/1741-2552/ad3678

**Published:** 2024-05-02

**Authors:** Nitin Sadras, Bijan Pesaran, Maryam M Shanechi

**Affiliations:** 1 Ming Hsieh Department of Electrical and Computer Engineering, Viterbi School of Engineering, University of Southern California, Los Angeles, CA, United States of America; 2 Department of Neurosurgery, Perelman School of Medicine, University of Pennsylvania, Philadelphia, PA, United States of America; 3 Thomas Lord Department of Computer Science, Alfred E. Mann Department of Biomedical Engineering, and the Neuroscience Graduate Program, University of Southern California, Los Angeles, CA, United States of America

**Keywords:** multimodal, spiking activity, local field potentials (LFP), neural decoding, maximum likelihood

## Abstract

The detection of events in time-series data is a common signal-processing problem. When the data can be modeled as a known template signal with an unknown delay in Gaussian noise, detection of the template signal can be done with a traditional matched filter. However, in many applications, the event of interest is represented in multimodal data consisting of both Gaussian and point-process time series. Neuroscience experiments, for example, can simultaneously record multimodal neural signals such as local field potentials (LFPs), which can be modeled as Gaussian, and neuronal spikes, which can be modeled as point processes. Currently, no method exists for event detection from such multimodal data, and as such our objective in this work is to develop a method to meet this need. Here we address this challenge by developing the multimodal event detector (MED) algorithm which simultaneously estimates event times and classes. To do this, we write a multimodal likelihood function for Gaussian and point-process observations and derive the associated maximum likelihood estimator of simultaneous event times and classes. We additionally introduce a cross-modal scaling parameter to account for model mismatch in real datasets. We validate this method in extensive simulations as well as in a neural spike-LFP dataset recorded during an eye-movement task, where the events of interest are eye movements with unknown times and directions. We show that the MED can successfully detect eye movement onset and classify eye movement direction. Further, the MED successfully combines information across data modalities, with multimodal performance exceeding unimodal performance. This method can facilitate applications such as the discovery of latent events in multimodal neural population activity and the development of brain-computer interfaces for naturalistic settings without constrained tasks or prior knowledge of event times.

## 1. Introduction

Event detection from time-series data is an important and well-studied signal-processing problem. When we observe time series data that contain a known event-related template signal with an unknown delay, the problem of event detection is to estimate this delay or event time. When the observed time series can be modeled as this known template signal in Gaussian noise, a traditional matched filter can be used to perform event detection [1, 2]. In various applications, however, the observed time series are multimodal, consisting of both Gaussian and point-process signals. In neuroscience experiments, for example, collected datasets can consist of simultaneous local field potentials (LFPs) and neuronal spiking activity, which can be modelled as Gaussian and point-process signals, respectively. While methods have been developed for event detection from each of these single modalities in isolation [2–5], no such method exists for multimodal time series containing a mixture of Gaussian and point-process signals.

A specific neuroscientific application of interest is that of brain-computer interfaces (BCIs), which are systems that aim to decode motor or cognitive states from a user’s neural activity to restore or augment task performance [6–25]. Prior work has shown that motor and cognitive states are represented across multiple spatiotemporal modalities of neural activity, and that BCI decoding of these states can significantly benefit from using multimodal data [26–45]. Such BCI decoders are often run on data where task-relevant event times, such as stimulus or movement onset, are known to the decoder, and data fed to the decoder can be time-locked to these known events. In many applications, however, it is possible that these event times are unknown, and as such time-locking cannot be performed during decoding. In order to enable high-performance multimodal motor or cognitive BCIs when blue either event times are unknown or tasks are not stereotyped, a method to simultaneously detect and decode events from multimodal signals blue in real time is necessary.

The problem of event detection from multimodal time series is challenging because of the differences in statistical properties of Gaussian and point-process signals [31, 46, 47] and because the events whose times are unknown can also have different yet unknown classes. Further, the spatiotemporal properties of the template signals corresponding to each event class must be known in order to perform detection and classification, so a learning method is required to fit these template signals. In order to address this challenge, we develop the multimodal event detector (MED), an algorithm which performs real-time event detection in multimodal time series with multiple unknown event classes. We do this by deriving the maximum-likelihood estimator of simultaneous event times and classes from multimodal data. We first write a parametric likelihood model of Gaussian and point-process time series that encode an event with an unknown time and class, develop a method to learn the model parameters from data, and then derive the estimate of the time and class that maximize this likelihood function. We validate this method in simulations and in spike-LFP neural datasets recorded from a nonhuman primate (NHP) performing a rapid eye-movement (saccade) task, with the goal of detecting the times and directions of saccades from continuous, un-epoched data. In simulated and real data, we show that the MED can successfully detect and classify eye movements from multimodal spike-field signals, using model parameters learned from training data. We further show that the MED successfully integrates information from both data modalities, with performance increasing as signal channels of either modality are added.

## 2. Methods

We first describe our model of how event times and classes are encoded in multimodal time series. We then derive the MED as the maximum-likelihood estimator of both event times and classes.

### 2.1. Multimodal model

Point process signals can be described as a time-series of 0s and 1s. These point processes can be characterized by a conditional intensity function (CIF) that models the rate *λ* at which nonzero values, or ‘spikes’, occur as a function of external covariates. We model point processes via a Poisson generalized linear model (GLM), where the logarithm of the CIF is a linear function of the covariates. Poisson point-process models have been successfully used to model neuronal spiking activity [48–59]. Further, prior work has developed Poisson GLMs that explicitly encode event times and classes, and showed that such models are a good fit to the spiking of neurons that encode stimulus-related events [60]. However, such models have not yet been used to develop multimodal detection and decoding algorithms, as we do here.

In the context of event detection, the covariates that we model are the event time $t_s \in [0, T] $ and the event class *s*, where $[0, T]$ is the interval on which the event can occur and *s* is a ‘one-hot’ vector in ${\mathbb{R}}^S$, where *S* is the number of possible classes. In a manner similar to [60], we write the point process CIF as

\begin{align*} \log \lambda_i\left(t\right) = r_i\left(t - t_s\right) \phi_i^Ts + \alpha_i. \end{align*}

Here, $\displaystyle \phi_i \in {\mathbb{R}}^S$ and $r_i(t)$ are, respectively, the spatial and temporal responses of channel *i*, while *α*_*i*_ characterizes the baseline rate. The spatial response encodes how a channel responds to the event class, while the temporal response encodes how a channel responds to the event time. The interpretation of this CIF is that an event at time *t_s_* will evoke a temporal response $r_i(t)$ that is modulated by a spatial response $\phi_i^Ts$. *φ*_*i*_ is a vector with the same size as *s*, and encodes the strength of channel *i*’s response to each event class. Given the CIF $\lambda_i(t)$ and assuming inhomogeneous Poisson statistics, the likelihood of observing a binary signal at channel *i* with spikes at times $\{t_{i,m}\}_{m = 1:M_i} = \{t_{i,0}, \ldots, t_{i, M_i}\}$ is given by

\begin{align*} p\left(\left\{t_{i,m}\right\}_{m = 1:{M_i}}\right) = e^{-Q_i}\prod_{m = 1}^{M_i} \lambda_i\left(t_{i,m}\right) \end{align*}

where $Q_i = \int_{0}^T \lambda_i(t) \, \mathrm{d}t$, *m* is the spike time index, and *M_i_* is the total number of spikes observed from channel *i* [61].

Similarly to our point process model, we model continuous channels as having spatial and temporal responses to an event at time *t_s_* of class *s*:

\begin{align*} y_j\left(t\right) = r_j\left(t - t_s\right) \phi_j^Ts + w_j\left(t\right) \, , \, w_j\left(t\right) \sim \mathcal{N}\left(0, \sigma_j^2\right) \end{align*}

where $w_j(t)$ is Gaussian noise and $\sigma_j^2$ is the variance of this noise for channel *j*. The corresponding likelihood is

\begin{align*} &amp;p\left({y_j\left(t\right)}_{t = 1:T}\right)\nonumber\\&amp;\quad = \prod_{t = 1}^T \frac{1}{\sqrt{2\pi\sigma_j^2}}\exp{-\frac{\left(y_j\left(t\right) - r_j\left(t - t_s\right)\phi_j^Ts\right)^2}{2\sigma_j^2}}. \end{align*}

In order to form a joint model of Gaussian and Poisson channels, we assume that they are conditionally independent, given the stimulus time *t_s_* and class *s*. Without this assumption, the number of parameters can become prohibitively large if interdependencies between every channel pair are considered, raising the possibility of overfitting to training data—especially in neural datasets that can be limited in sample size. Prior studies have verified that it is reasonable to assume conditional independence between LFPs and spikes (e.g. [31, 35]), and as such, we use this assumption in order to keep our model and estimators tractable and not prone to overfitting.

With this assumption, the joint likelihood for *I* Poisson channels and *J* Gaussian channels is

\begin{align*} \mathcal{L}\left(t_s, s\right) &amp;= {} p\left(\left\{t_{i,m}\right\}_{\substack{i = 1:I\\m = 1:M_i}}, \left\{y_j\left(t\right)\right\}_{\substack{j = 1:J\\t = 1:T}} \, | \, t_s, s\right) \\ &amp;= {} \prod_{i = 1}^I p\left(\left\{t_{i,m}\right\}_{\substack{i = 1:I\\m = 1:M_i}} \, | \, t_s, s\right)\nonumber\\ &amp;\quad \times \prod_{j = 1}^J p\left(\left\{y_j\left(t\right)\right\}_{\substack{j = 1:J\\t = 1:T}} \, | \, t_s, s\right). \end{align*}

Substituting in our likelihood models from (2) and (4), we get

\begin{align*} \mathcal{L}\left(t_s, s\right) &amp;= {} \prod_{i = 1}^I e^{-Q_i}\prod_{m = 1}^{M_i} \exp\left(r_i\left(t_{i,m} - t_s\right) \phi_i^Ts + \alpha_i\right) \nonumber\\ &amp;\quad\times \prod_{j = 1}^J \prod_{t = 1}^T \frac{1}{\sqrt{2\pi\sigma_j^2}}\nonumber\\ &amp;\quad\times\exp \left(-\frac{\left(y_j\left(t\right) - r_j\left(t - t_s\right)\phi_j^Ts\right)^2}{2\sigma_j^2}\right). \end{align*}

This provides a multimodal model of Gaussian and point-process signals that encode both event times, via a temporal response, and event classes, via a spatial response. We note that prior work has been done to model spatiotemporal responses for point process signals [5, 60], but the above does so for multimodal signals to solve the unaddressed problem of simultaneous event detection and classification from multimodal signals, as we do next.

### 2.2. Maximum likelihood estimate of event times and classes

Using the models defined in section 2.1, we can now formalize the problem of simultaneous event detection and classification as an optimization problem. Specifically, our goal is to find the event time and class that maximize the log-likelihood of the observed multimodal data:

\begin{align*} \left(\hat{t_s}, \hat{s}\right) &amp;= {} \arg \max_{\!\!\!\!\!\!\!\!\!\!\!\!\!\!\!\!t_s, s} \log \mathcal{L}\left(t_s, s\right) = {} \arg \max_{\!\!\!\!\!\!\!\!\!\!\!\!\!\!\!\!t_s, s} \sum_{i = 1}^I -Q_i\left(s\right)\nonumber\\ &amp;\quad + \sum_{m = 1}^{M_i} \left(r_i\left(t_{i,m} - t_s\right) \phi_i^Ts + \alpha_i\right) \nonumber\\ &amp;\quad+ \sum_{j = 1}^J \sum_{t = 1}^T -\frac{1}{2}\log\left(2\pi\sigma_j^2\right)\nonumber\\ &amp;\quad - \frac{1}{2\sigma_j^2}\left(y_j\left(t\right) - r_j\left(t-t_s\right)\phi_j^Ts\right)^2. \end{align*}

Removing additive terms that do not vary with *t_s_* or *s*, and therefore do not impact the argmax, this simplifies to:

\begin{align*} \left(\hat{t_s}, \hat{s}\right) &amp;= {} \arg \max_{\!\!\!\!\!\!\!\!\!\!\!\!\!\!\!\!t_s, s} \sum_{i = 1}^I -Q_i\left(s\right) + \sum_{m = 1}^{M_i} r_i\left(t_{i,m} - t_s\right) \phi_i^Ts \nonumber\\ &amp;\quad+ \sum_{j = 1}^J \sum_{t = 1}^T \frac{1}{\sigma_j^2}\left(y_j\left(t\right)r_j\left(t-t_s\right)\phi_j^Ts\right. \nonumber\\ &amp;\quad\left. - \frac{1}{2}\left(r_j\left(t-t_s\right)\phi_j^Ts\right)^2 \right). \end{align*}

To further simplify this expression, we define $u_i(t)$ as the Poisson binary time series:

\begin{align*} u_i\left(t\right) = \sum_{m = 1}^{M_i}\delta\left(t - t_{i, m}\right) \end{align*}

where $\delta(t)$ is the Dirac delta function. We can now re-write the following terms from (6) as convolutions:

\begin{align*} \sum_{m = 1}^{M_i} r_i\left(t_{i,m} - t_s\right) \phi_i^Ts &amp; = \sum_{t = -\infty}^{\infty} u_i\left(t\right) r_i\left(t - t_s\right) \phi_i^Ts\\ &amp; = \phi_i^Ts\left(u_i\left(t_s\right) * r_i\left(-t_s\right) \right) \end{align*}

and

\begin{align*} \sum_{t = 1}^T y_j\left(t\right)r_j\left(t-t_s\right)\phi_j^Ts &amp; = \phi_j^Ts\left(y_j\left(t_s\right) * r_j\left(-t_s\right) \right) \end{align*}

where $*$ represents convolution. Substituting these convolution expressions into (6), we get

\begin{align*} \left(\hat{t_s}, \hat{s}\right) &amp;= {} \arg \max_{\!\!\!\!\!\!\!\!\!\!\!\!\!\!\!\!t_s, s} \sum_{i = 1}^I \phi_i^Ts\left(u_i\left(t_s\right) * r_i\left(-t_s\right)\right) -Q_i\left(s\right)\nonumber\\ &amp;\quad+ \sum_{j = 1}^J \frac{1}{\sigma_j^2}\left(\vphantom{\frac{1}{2} \sum_{t = 1}^T \left(r_j\left(t-t_s\right)\phi_j^Ts\right)^2} \phi_j^Ts \left(y_j\left(t_s\right) * r_j\left(-t_s\right) \right)\right.\nonumber\\ &amp;\quad\left. - \frac{1}{2} \sum_{t = 1}^T \left(r_j\left(t-t_s\right)\phi_j^Ts\right)^2 \right). \end{align*}

Finally, we note that if the support of *r*(*t*) is much smaller than *T*, then the term $\sum_{t = 1}^T r_j(t-t_s)$ does not vary with *t_s_*. This is because delaying $r_j(t)$ by *t_s_* samples does not change the value of its summation over the entire duration *T*, as long as the entire support of $r_j(t - t_s)$ lies within $[0,T]$. Assuming that this is true, we can further simplify the second term in the sum over the *J* Gaussian channels in (8):

\begin{align*} \left(\hat{t_s}, \hat{s}\right) &amp;= {} \arg \max_{\!\!\!\!\!\!\!\!\!\!\!\!\!\!\!\!t_s, s} \sum_{i = 1}^I \phi_i^Ts\left(u_i\left(t_s\right) * r_i\left(-t_s\right)\right) -Q_i\left(s\right) \nonumber\\ &amp;\quad+ \sum_{j = 1}^J \frac{1}{\sigma_j^2}\left(\vphantom{\frac{\left(\phi_j^Ts\right)^2}{2} \sum_{t = 1}^T r^2_j\left(t\right)}\phi_j^Ts \left(y_j\left(t_s\right) * r_j\left(-t_s\right) \right)\right.\nonumber\\ &amp;\quad\left. - \frac{\left(\phi_j^Ts\right)^2}{2} \sum_{t = 1}^T r^2_j\left(t\right) \right). \end{align*}

This is a sum of linear matched filters that are matched to the temporal responses *r*(*t*) and scaled by the spatial responses $\phi^Ts$. Point processing channels are vertically shifted by the term $Q_i(s)$, while Gaussian channels are vertically shifted by $\frac{(\phi_j^Ts)^2}{2} \sum_{t = 1}^T r^2_j(t)$ and then scaled by their inverse noise variance $\sigma_j^2$. The shift terms set the baseline levels for each channel’s contribution to the MED output. The inverse noise variance scaling for Gaussian channels means that noisier channels have a smaller contribution to the MED output.

An additional challenge in multimodal integration when some modalities are discrete and some are continuous is that of proper scaling of likelihoods. In particular, while the point process likelihood for discrete random variables provides a probability measure (i.e. probability of a number of spikes), the Gaussian likelihood for continuous random variables provides a density measure that needs to be integrated over a range of values to provide a probability measure (in and of itself is not a probability). This distinction between discrete and continuous modalities makes the multimodal approach sensitive to scaling of the continuous signals (see details in section 4). To address this challenge and additionally account for model mismatch in real datasets, we introduce a single cross-modal scaling parameter *k* that weighs the contributions of the two data modalities in the likelihood model:

\begin{align*} \left(\hat{t_s}, \hat{s}\right) &amp;= {} \arg \max_{\!\!\!\!\!\!\!\!\!\!\!\!\!\!\!\!t_s, s} \sum_{i = 1}^I \phi_i^Ts\left(u_i\left(t_s\right) * r_i\left(-t_s\right)\right) -Q_i\left(s\right) \nonumber\\ &amp;\quad+ k\sum_{j = 1}^J \frac{1}{\sigma_j^2}\left(\vphantom{\frac{\left(\phi_j^Ts\right)^2}{2} \sum_{t = 1}^T r^2_j\left(t\right)} \phi_j^Ts \left(y_j\left(t_s\right) * r_j\left(-t_s\right) \right)\right. \nonumber\\ &amp;\quad\left.- \frac{\left(\phi_j^Ts\right)^2}{2} \sum_{t = 1}^T r^2_j\left(t\right) \right). \end{align*}

This scaling parameter can be learned via grid-search on training data by maximizing any performance metric of interest. Here, we choose the value of *k* that maximizes the sum of the event detection area under this curve (AUC) (see section 2.5) and event classification accuracy on training data.

**Figure 1.**
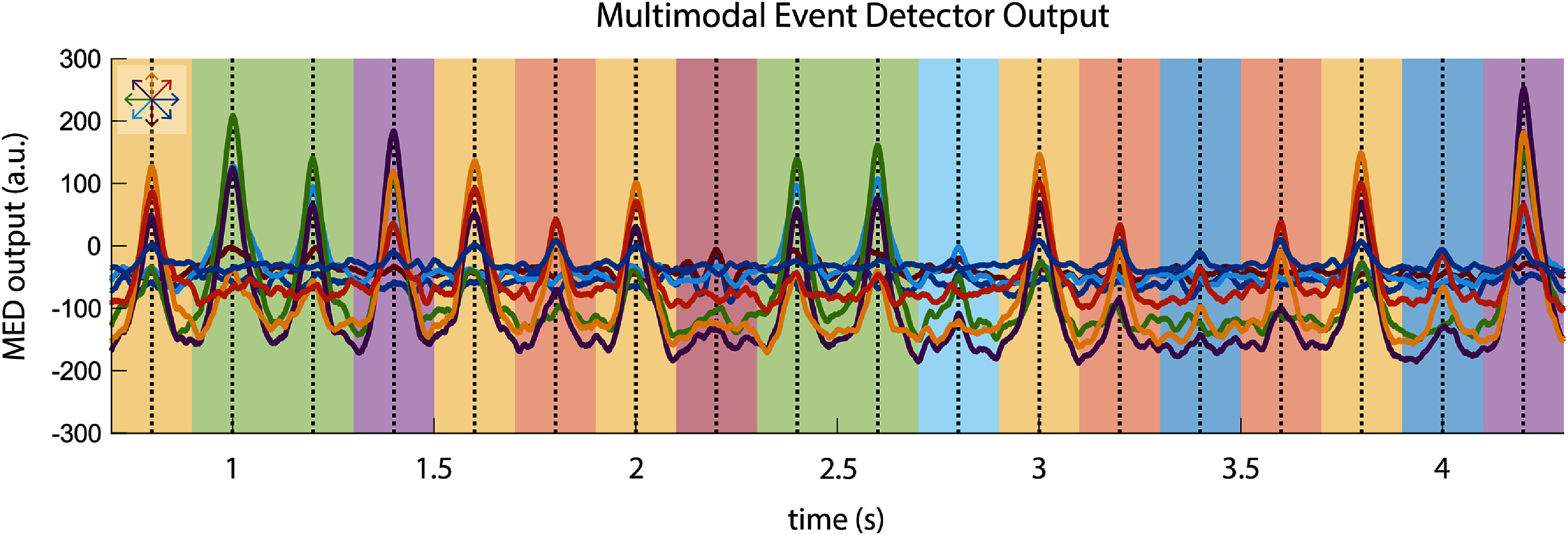
Sample output of the MED on simulated multimodal neural activity. In this example, the events are rapid eye movements (saccades), and the event classes are one of 8 eye movement directions. The MED produces 8 output signals, one for each class, which are shown plotted here. The color of the background indicates the true saccade direction at a given time, while the color of the maximum-valued output signal corresponds to the maximum-likelihood estimate of the saccade direction. The true saccade times are indicated by vertical dashed lines, while peaks in the maximum-valued output signal correspond to the maximum-likelihood estimate of the saccade times. The mapping between colors and saccade directions is shown in the top left. In this example, we can see that the MED output peaks align with the true saccade times, while the colors of the peak valued signals match the background, indicating that the MED is successfully detecting and classifying saccades from the simulated data.

We can interpret the output of the MED as *S* separate signals, or one for each possible event class. At any given time, the maximum-valued output signal corresponds to the maximum-likelihood estimate of the event class, while the peaks in this maximum-valued signal correspond to the maximum-likelihood estimates of the event time. This idea is illustrated using simulated spike-field data in figure 1, where correct saccade classification is indicated by the background color matching the color of the peak signal.

We note that if the temporal responses *r*(*t*) have finite support, the sum of matched filters in (10) can be computed causally, with a fixed delay. Specifically, if the *r*(*t*) equals zero outside of the interval $[t_1, t_2]$, with *t* = 0 indicating the event time, and if $t_2> 0$, we can delay the matched filter kernels $r(-t)$ by *t*_2_ seconds in order to compute the convolutions causally. This would cause peaks in the MED output to occur *t*_2_ seconds after the corresponding event. Intuitively, this delay is necessary because the neural response to an event at time *t* = 0 will last until time $t = t_2$, and we must observe the entire duration of the neural response to accurately decide if an event has occurred or not. Any latency caused by real-time pre-processing would further increase this delay.

In order to use the MED in a real dataset, the parameters of our model in (5) must be estimated from training data. A maximum-likelihood method for estimating these parameters is described in appendix A.

### 2.3. Multimodal model of saccade-sensitive neural activity

**Figure 2.**
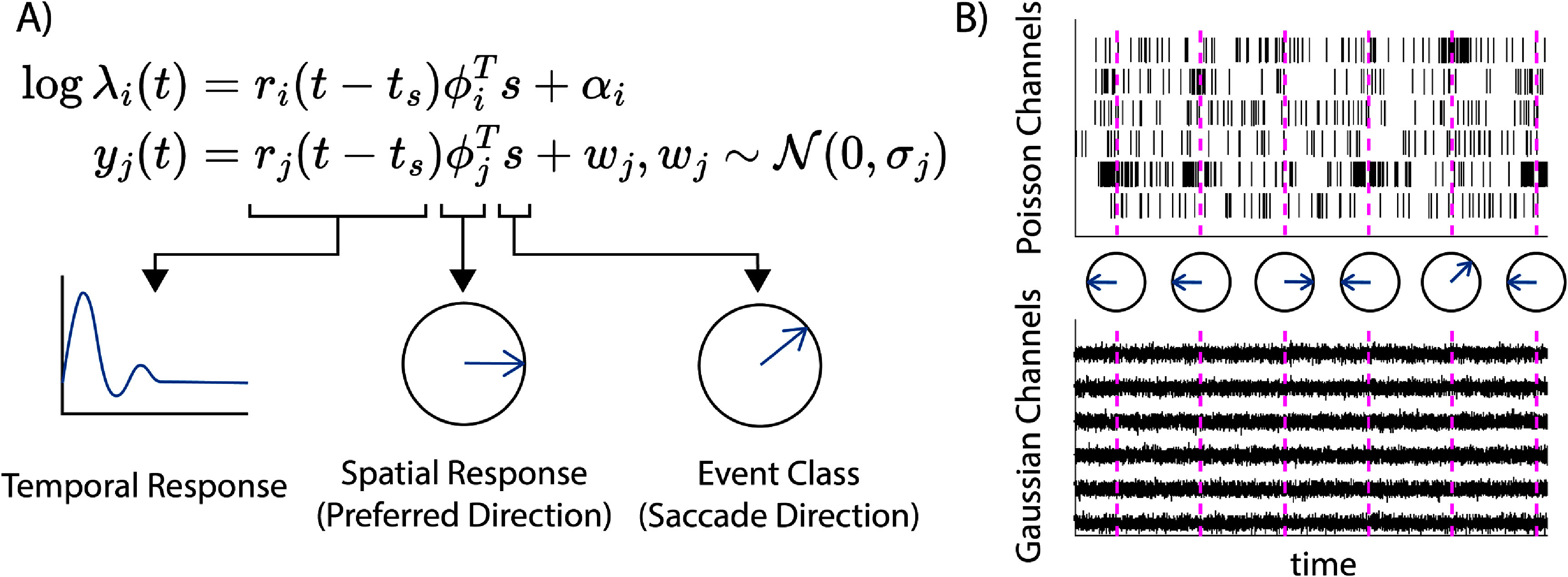
Model of saccade-sensitive multimodal neural activity. (A) *r*(*t*) is the temporal response of the channel to an event at time *t_s_*, while the spatial parameter *φ* encodes the magnitude of the channel’s response to each possible saccade direction *s*. (B) Simulated Poisson (top) and Gaussian (bottom) data based on the model in A. Saccade times are indicated by vertical dashed lines, and the corresponding saccade directions are indicated by the circled arrows.

Prior work has shown that saccadic eye movements elicit transient responses in neural activity that depend on the saccade direction [5, 60]. This provides an ideal validation setting for the MED by allowing us to test whether it can be used to detect and classify saccades from multimodal neural activity. In the dataset described in section 2.4, there are 8 possible eye movement targets, and as such we consider the case where there are eight possible saccade directions. We model this by making our event class *s* a one-hot vector in $\displaystyle {\mathbb{R}}^8$, with each entry representing one possible saccade direction. The spatial responses $\phi \in \displaystyle {\mathbb{R}}^8$ encode how saccades to each possible direction change the magnitude of the temporal response *r*(*t*). For example, if channel *i* has a strong response to the 1st saccade direction (i.e. its ‘preferred direction’ is direction 1), then the first element of *φ*_*i*_ would be large, while the remaining seven would be smaller. This saccade encoding model is illustrated in figure 2(A). Simulated multimodal data based on this model is shown in figure 2(B).

### 2.4. NHP saccade task

In addition to simulations, we validated the MED on multimodal spike-LFP data collected from the prefrontal cortex (PFC) of an NHP performing a delayed saccade task. The task design is as follows. First, the NHP was required to maintain its gaze on a central point on a screen for 500–800 ms. After this fixation, a target cue appeared in one of eight possible locations. After a delay period of 1000–1500 ms, a ‘go’ cue prompted the NHP to make a saccade to the target. A movable electrode array consisting of 32 electrodes (Gray Matter Research, USA) was placed over the prearcuate gyrus of the lateral PFC to record neural activity. Raw neural signals were sampled at 30 kHz. In order to isolate single-unit activity, the raw data was preprocessed by high-pass filtering at 300 Hz and thresholding at 3.5 standard deviations below the signal mean. Spike sorting was then performed via principal component analysis and k-means clustering. LFP signals were acquired by band-pass filtering the raw data from 0.5 to 300 Hz. Both spike and LFP signals were then downsampled to 1 kHz. Previous LFP studies have used similar preprocessing pipelines [28, 39, 62–66]. This minimal preprocessing pipeline also has the benefit of reducing the latency of the MED’s output, as discussed in section 2.2. Eye position was recorded using an infrared eye-tracking system (ISCAN, USA) with a sampling rate of 120 Hz. All surgical and experimental procedures were in compliance with National Institute of Health Guide for Care and Use of Laboratory Animals and were approved by the New York University Institutional Animal Care and Use Committee. Further details can be found in [65].

### 2.5. Performance evaluation

We assess the ability of the MED to detect event times using receiver operating characteristic (ROC) curve analysis [67]. The ROC plots the probability of true detection of events against the probability of false detection as a threshold for detection is varied. The AUC is used as a threshold-free performance metric, with an AUC of 0.5 indicating chance-level detection and an AUC of 1 indicating perfect detection. We use a modified version of the AUC that counts detections that are within 200 ms of a true event to count as true detections.

The details of our modified AUC measure are as follows. Let *x*(*t*) be the maximum value of the MED’s output at any time *t*, and let *h* be an event detection threshold. We consider all peaks in *x*(*t*) that are above the threshold *h*. If a peak is within 200 ms of an actual saccade, then it is considered a true positive. If it is not, then it is considered a false positive. In this way, we record the true positive rates (TPRs) and false positive rates (FPRs) as the threshold *h* varies from $\min(x(t))$ to $\max(x(t))$. We can then plot these FPR and TPR values against each other to construct an ROC curve, and the AUC is our AUC metric.

## 3. Results

We validated the MED in the context of saccade detection from neural activity, using both numerical simulations and NHP neural signals. In both cases, the goal of the MED is to detect saccade onset time and direction.

### 3.1. Simulation results

We simulated the activity of five Poisson and five Gaussian channels using the model described in section 2.3. We generated neural signals corresponding to 20 saccades, with a jittered 2 second gap between each saccade. The simulated point process channel signals had a minimum firing rate of 10 Hz and a maximum firing rate of 100 Hz, while Gaussian signals had an signal-to-noise ratio (SNR) of 0.1. These values were chosen in order to roughly match the properties of the real dataset we analyze in section 3.2. We repeated this simulation 104 times and for each repetition the true neural parameters, saccade directions, and saccade time jitters were randomized. The true neural parameters were used to generate training and test data for each iteration, and neural parameters were estimated from the training data using the method described in appendix A. These estimated parameters were then used to perform simultaneous saccade time detection and direction classification on the test data.

**Figure 3.**
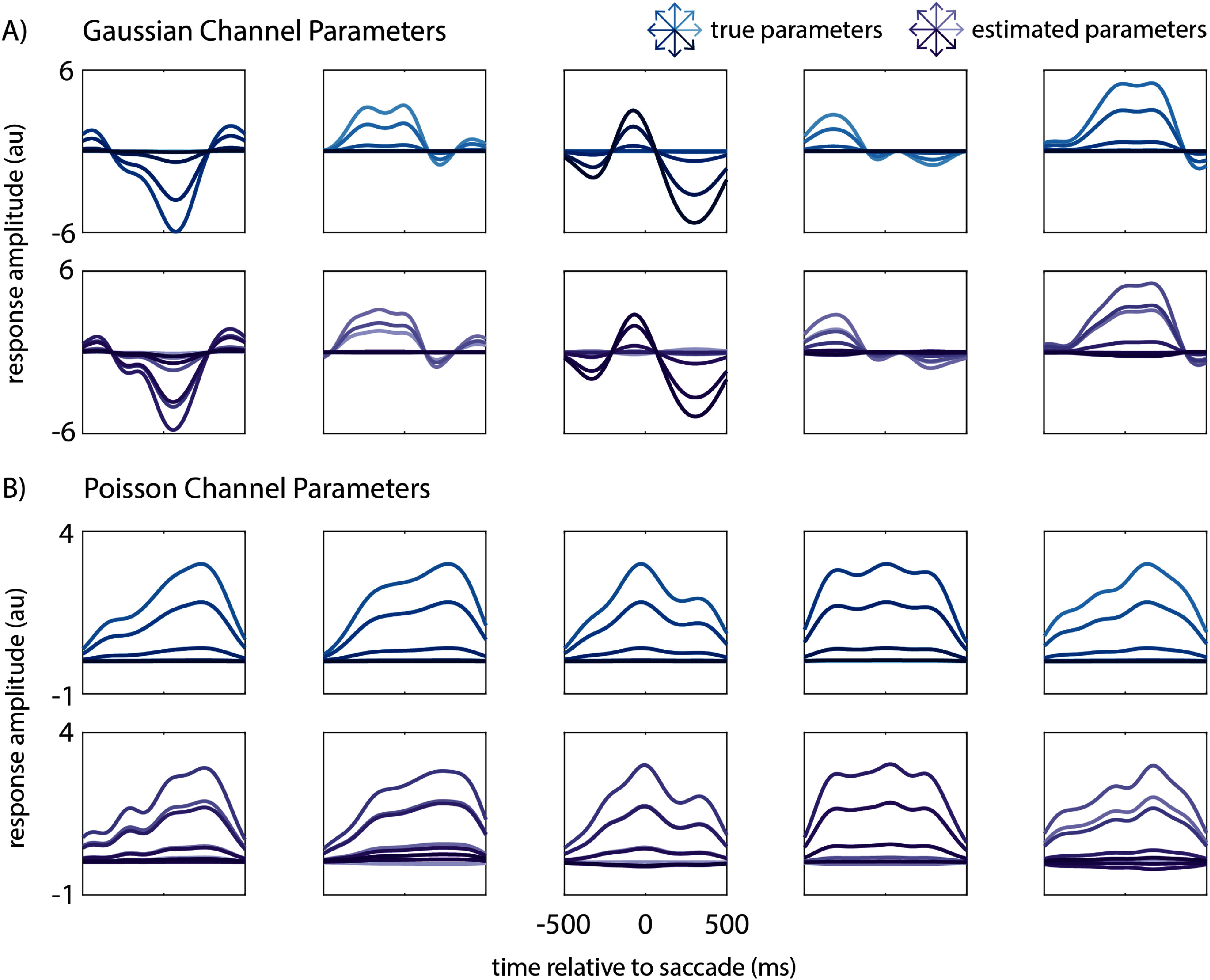
Comparison of true and estimated parameters based on simulated multimodal data. We simulated multimodal neural activity that encodes saccade time and direction, and used maximum likelihood estimation to estimate the model parameters. (A) Ground truth (top row) and estimated (bottom row) spatiotemporal responses for five example Gaussian channels. Each trace indicates the channel’s response to a specific saccade direction, indicated by its color. The mapping between trace colors and saccade directions is shown on the top right. Our maximum likelihood estimation procedure resulted in parameters that closely match the ground truth. (B) Same as A but for five example Poisson channels.

**Figure 4.**
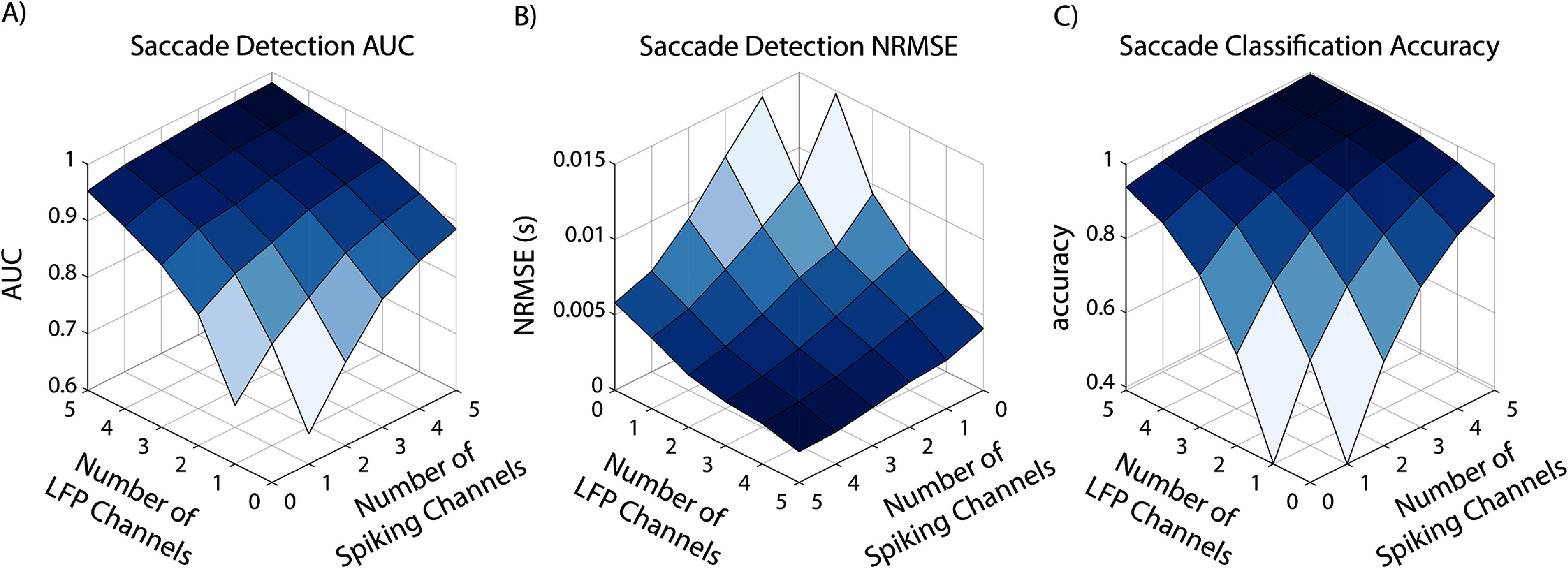
MED performance on simulated saccade-sensitive multimodal neural activity. All measures are shown as a function of the number of channels of each modality and are averaged over 104 simulation repetitions. (A) Saccade time detection area under the curve (AUC); higher AUC indicates better saccade detection performance. (B) Saccade time detection normalized root-mean-square error (NRMSE); lower NRMSE indicates better performance. (C) Saccade direction classification accuracy. The MED successfully combines information across data modalities, with performance increasing monotonically as channels of either type are added.

A comparison of the estimated parameters with the ground truth is shown in figure 3. The event detection and classification performance metrics, averaged across simulation iterations and as a function of multimodal channel counts, are shown in figure 4. This simulation analysis shows that: (1) we can successfully estimate spatial and temporal response parameters from simulated multimodal data and (2) the MED can use these estimated parameters to successfully perform simultaneous event detection and classification from multimodal data. Importantly, the MED successfully combines information across data modalities, with performance increasing monotonically as channels of either modality are added.

In order to investigate the MED’s ability to handle increasingly noisy signals, we repeated this simulation analysis for varying levels of spike and LFP noise. The results of this analysis are shown in appendix B.

### 3.2. NHP data results

To validate the MED on a real-world dataset, we used spike-LFP neural data recorded from an NHP performing a delayed-saccade task. In this task, detailed in section 2.4, the NHP first had to maintain its gaze on a central fixation point on a screen, and then make a saccade to one of eight peripheral targets after a ‘go’ cue [65]. We used a subset of this data consisting of eight continuous recording sessions (average: 233.875 trials/session, total: 1871 trials). We evaluated the MED via leave-one-session-out cross validation over the eight sessions, where all sessions except for one were used to train the MED, and the remaining session was used to test it. This cross-validation was repeated eight times so that each session could be used for testing. We performed cross validation over sessions in order to evaluate the MED’s performance on a continuous stream of un-epoched data (as opposed to individual trials), as would be the case in a real-time application.

In order to explore how the MED integrates information across modalities, we measured performance as channels of both modalities were added one at a time. For each cross-validation fold, we shuffled the order in which spike and LFP channels were added 10 times. These 10 shuffles were the same for each fold.

**Figure 5.**
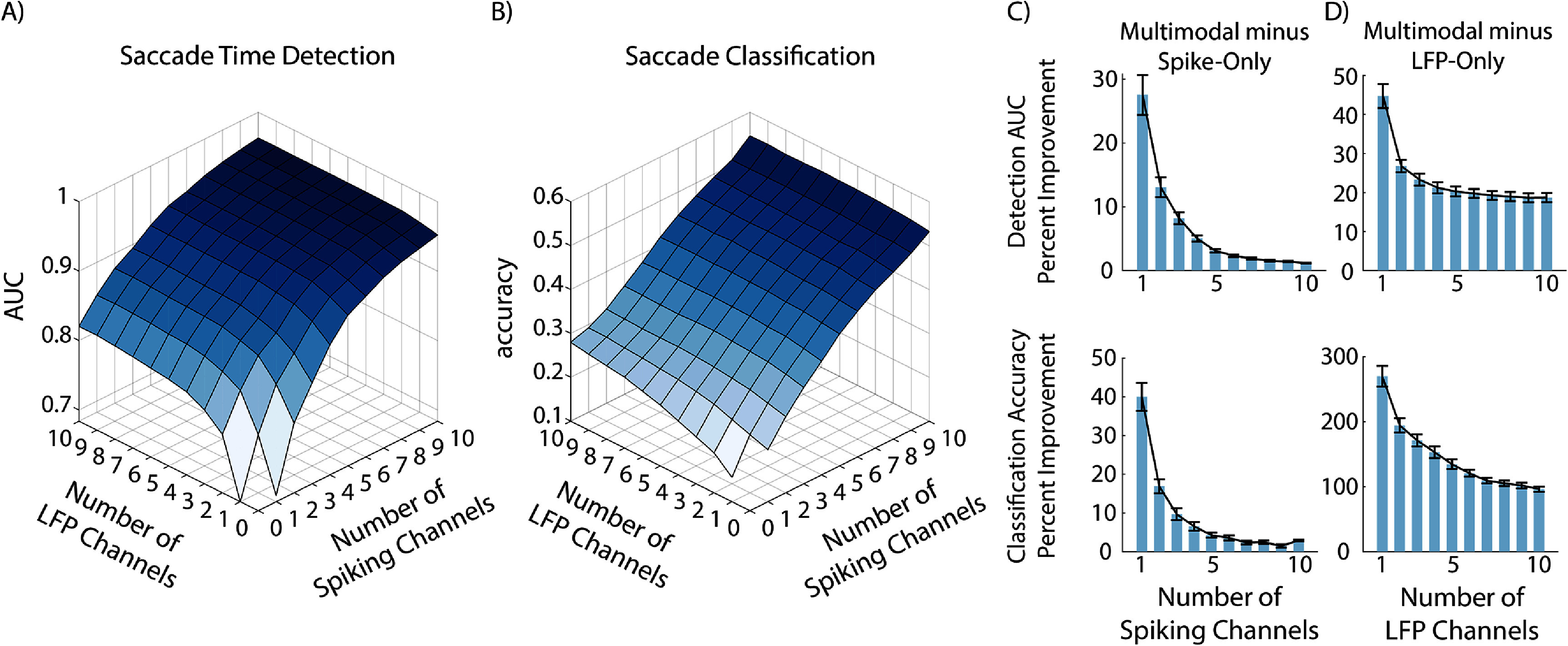
MED performance on multimodal nonhuman primate (NHP) neural data. (A) Saccade time detection AUC. (B) Saccade direction classification accuracy. Results in A and B are averaged over 8 cross-validation folds and 10 shuffles of channel order. (C) Performance benefit of using multimodal data over spike-only data, as a function of the number of spike channels. Performance benefit is shown for both saccade time detection AUC (top) and direction classification accuracy (bottom). Bars indicate the mean percent improvement from spike-only to multimodal performance, computed as $100*(\text{multimodal performance - }$ $\text{unimodal performance})/(\text{unimodal performance}$), and error bars indicate the standard error of the mean (SEM). Multimodal data includes all LFP channels, with different number of spike channels (i.e. spike count) as indicated on the *x*-axis. For all spike-count values, there was significant benefit in adding LFP channels (*p* $\lt$ .001, paired *t*-test, *N* = 80). (D) Same as C, but showing the benefit of using multimodal data over LFP-only data. For all LFP-count values, there was significant benefit in adding spike channels (*p* $< 1\times 10^{-14}$, paired *t*-test, *N* = 80). The reported *p*-values for panels (C) and (D) are corrected for multiple comparisons within each plot via the Benjamini–Hochberg procedure—specifically, the 10 *p*-values in each plot, one per bar, are used as input to the procedure.

The MED was able to successfully detect and classify saccades simultaneously from multimodal spike-LFP data. First, even with a relatively low number (10) of spike and LFP channels, the MED achieved a saccade time detection AUC of 0.96 (chance level = 0.5) and a saccade direction classification accuracy of 0.55 (chance level = 0.125). These are both significantly higher ($p < 5\times 10^{-5}$, paired *t*-test, *N* = 80) than the corresponding chance levels. Second, the MED successfully achieved multimodal fusion, with multimodal performance exceeding unimodal performance. Both detection AUC and classification accuracy increased as channels of either type were added, as shown in figures 5(A) and (B). Indeed, multimodal performance was significantly greater than both spike-only (*p* $< 1\times 10^{-3}$, Hochberg-corrected paired t-test, N = 80) and LFP-only performance (p $< 1\times 10^{-14}$, Hochberg-corrected paired *t*-test, *N* = 80) for all unimodal channel counts, as shown in figures 5(C) and (D). We used the Benjamini–Hochberg procedure to control the false discovery rate of the t-tests within each plot in figures 5(C) and (D). Specifically, for each plot, the 10 *p*-values (one per bar) corresponding to that plot were used as input to the correction procedure. While this performance increase was indeed significant for all unimodal channel counts, the increase was larger in the low-information regime, i.e. when unimodal channel counts were smaller. We note that the true efficacy of the MED is demonstrated by the fact that multimodal performance is significantly greater than unimodal performance, as shown in figures 5(C) and (D).

Interestingly, despite LFP-only performance being lower than spike-only performance, the MED was still able to improve upon spike-only performance by adding LFPs. This indicates that the MED truly integrated information from both modalities, taking advantage of information present in LFP channels that was not present in spike channels. This result also suggests that spiking and LFP activities carry non-redundant information about both the timing and the class of a saccade event.

## 4. Discussion

In this work we developed the MED, which solves the unaddressed problem of event detection and classification from multimodal point-process and Gaussian time series. We showed that both in simulated data and in an NHP neural dataset, the MED was able to simultaneously detect and classify eye movements in a way that successfully combined information across data modalities.

### 4.1. Bi-directional performance improvement

A successful multimodal decoder should take advantage of the information present in all available data modalities. To show that the MED does this, we performed an extensive performance analysis in which channels of both modalities were incrementally included as input to the MED. On both simulated and real NHP data, this analysis showed that MED performance improves bidirectionally—both when LFP channels are added to a fixed number of spike channels, and when spike channels are added to a fixed number of LFP channels. In the NHP dataset, although spike-only performance was generally better than LFP-only performance, multimodal performance was still significantly better than both. These results suggest that LFPs contain information about both event timing and event class that is not present in spikes, and vice versa.

### 4.2. Cross-modal scaling

As mentioned in section 2.2, we modified the maximum-likelihood estimator of event times and classes by adding a cross-scale combination parameter. As we explained, this is necessary when we have a combination of discrete and continuous modalities, because the likelihood in the former provides a probability measure while the likelihood in the latter requires integration over a range of values to give a probability. Thus combining likelihoods of discrete and continuous modalities is sensitive to scalings of the latter. Indeed, scaling a Gaussian signal will in turn scale its likelihood, even though its SNR is unchanged.

To see why this is, consider a signal $y = s + n$, where *s* = *b* is a constant signal and $n \sim \mathcal{N}(0, \sigma^2)$ is noise. The SNR of this signal is $b^2/\sigma^2$, and the likelihood of observing *y* = *b* is given by the peak of the Gaussian PDF $1/(\sigma\sqrt{2\pi})$. If *b* = 1 and *σ* ^2^ = 1, then the SNR of *y* is 1 and the likelihood of observing *y* = 1 is $ 1/(\sqrt{2\pi}) \approx 0.4$. If we then multiply *y* by 2, this is equivalent to setting *b* = 2 and *σ* ^2^ = 4. The SNR is still 1, but the likelihood of observing *y* = 2 is now $1/(2\sqrt{2\pi}) \approx 0.2$. So, even though scaling *y* does not change its SNR, it *does* change its likelihood. This is problematic because ideally the contribution of Gaussian channels to the MED output should depend only on their SNR, and not on their scale. In practice, we found that a single parameter weighing the contribution of all Gaussian channels together was sufficient to properly combine their information with the point-process channels, thereby alleviating this issue.

In addition to the above mathematical reason, another practical reason for using this scaling parameter is model mismatch in real neural datasets. If any of the model parameters are incorrectly estimated in a systematic way across a single modality, then the learned model will not properly weigh the contributions of each modality. For example, it is possible that there are events unrelated to eye movements that also elicit spatiotemporal responses in both types of channels. For Gaussian channels, this can be accounted for by the noise variance parameter *σ*, but Poisson models do not have a way to account for this kind of variance. This phenomenon could cause one scale to be ignored even if it has information to contribute.

### 4.3. Applications and future directions

In section 2.2, we assumed conditional independence between the spiking and LFP modalities, conditioned on event times and classes, in order to make the derivations tractable. Since the MED successfully combined information across data modalities in both event detection and classification, we can conclude that this is a reasonable assumption. Nevertheless, future work can consider modeling such interdependencies while regularizing or sparsifying them to avoid overfitting, and explore whether such modeling leads to improved performance.

The MED also makes a simplifying assumption that LFP noise is Gaussian, zero-mean, and stationary. These assumptions allowed us to derive a tractable estimator with relatively few parameters. Further, the MED performed well on our NHP dataset, indicating that with these assumptions, our model works successfully for the task of multimodal event detection. While the LFP signals may not be strictly Gaussian, this Gaussian model has been successful in many prior studies [26, 30, 31, 35, 39, 63, 64, 68] as well as here, showing its utility for various applications. However, neural data statistics can change over time, especially in longer recordings due to various factors such as electrode impedance changes or neural plasticity causing changes in the signal statistics [69–73]. In order to deal with non-stationary signals, adaptive decoding algorithms that can track these nonstationarities are needed. Indeed, prior work has developed methods that can estimate and update model parameters in real time, though these approaches have largely focused on single modalities and on the task of decoding rather than event detection [9, 10, 57, 74–77]. Future work can extend prior adaptive methods for event detection and multimodal signals so that they can be used with the MED. For example, one simple approach for using MED in situations with non-stationary neural statistics is to retrain the model intermittently using the method described in the appendix. This could allow the MED to maintain performance over longer periods of time.

The development of deep-learning event detection and classification methods is also a promising future direction. Although the MED performed well on our saccade dataset, it is possible that deep-learning methods, such as convolutional neural networks (CNNs), may improve detection performance for certain neural populations and tasks. For example, unimodal CNNs have been used in the field of astrophysics to successfully detect black hole merging events from unimodal gravitational wave signals [78, 79]. However, such CNNs have not yet been developed for multimodal data or for neural data whether unimodal or multimodal. Further, they have not been developed or tested for the classification of multiple event types, which is what we must do here as our events have not only unknown times, but also unknown classes. As such, the development of a multimodal deep learning method for event detection, and an investigation of the circumstances under which it can outperform alternative methods, is an interesting direction of future research.

Lastly, the application of the MED in a real-time BCI is an important line of research. While motor BCIs to date have focused on continuous movement decoding, the development of cognitive BCIs will in many contexts require event detection in order to perform decoding. For example, in the context of a stimulus-based decision task, one would first need to detect a stimulus onset in order to then decode decision-related information, such as the decision itself or the associated confidence [80–83]. Thus, the MED can enable multimodal cognitive BCIs for decision making in real-world applications where event times are unknown. The MED can also help extend multimodal motor BCIs to naturalistic setups where task-related events, such as movement onset, must be detected before decoding is possible. In addition to spiking and LFP, the application of MED to other neural modalities such as intracranial EEG or electrocorticography, which have high potential for translation, is also an important future direction [22, 74, 75, 84–96]. Finally, the combination of MED with various learning algorithms for neural population modeling and decoding, even in the presence of external input stimuli (e.g. [19, 91, 97, 98]), is an interesting future direction.

## Figures and Tables

**Figure B1.**
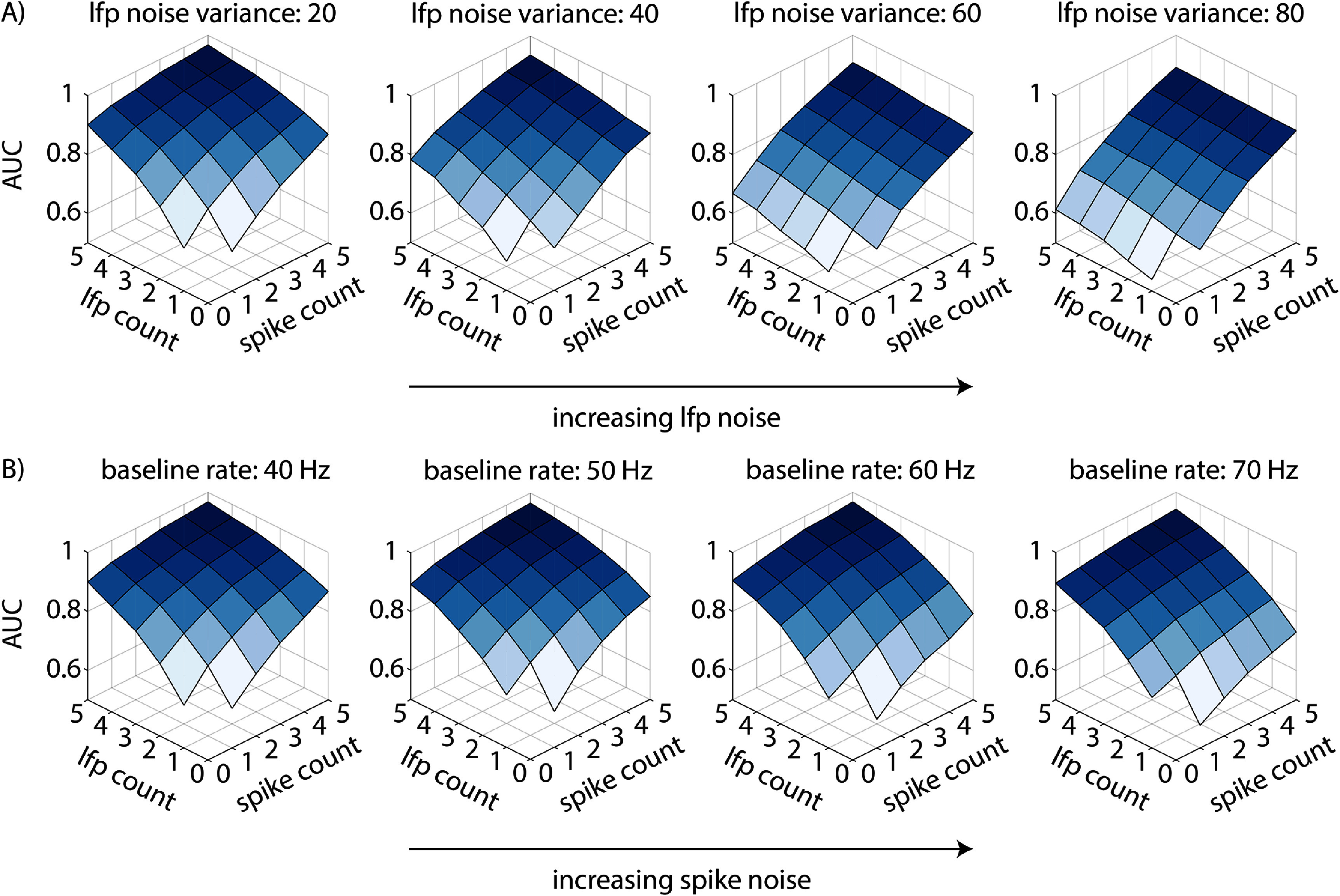
MED noise sensitivity analysis. We repeated the simulation analysis shown in figure 4 while varying the noise statistics. (A) MED saccade detection AUC as LFP noise variance is increased from 20 to 80 units. The maximum LFP signal amplitude is fixed at 1 unit, and the baseline firing rate for spikes is fixed at 40 Hz. (B) Saccade detection AUC as spike baseline firing rate is increased. The peak firing rate of the spike temporal responses was fixed at 100 Hz, so that increasing the baseline firing rate would make it harder to detect events. The LFP noise variance is fixed at 20 units. For both (A) and (B), even though LFP-only and spike-only performance decline as their respective noise levels are increased, the MED is still able to combine information from both modalities and increase performance as channels of either type are added.

## Data Availability

The main data supporting the results are available within the paper. The raw data is too large to be hosted and shared publicly. The data that support the findings of this study are available upon reasonable request from the authors.

## Appendix A Model parameter estimation

In order to make use of the MED, the model parameters of each channel must be estimated. Prior work has developed a way to estimate spatial and temporal parameters for Poisson GLMs [60]. This method can also be used with Gaussian linear models. Building on this prior work [60] for Poisson GLMs, we estimate the parameters in our multimodal model as follows.

The parameters that we must estimate are the temporal responses *r*(*t*), the spatial responses *φ*, the Poisson baseline parameters *α*_*i*_, and the Gaussian noise variances *σ*_*j*_. We can estimate these parameters by maximizing the likelihood of a training dataset over these parameters. For Poisson signals, this can be done efficiently via iteratively re-weighted least squares, and for Gaussian signals, this can be done via simple linear regression. However, these methods require that our models are log-linear in the parameters for Poisson channels, and linear in the parameters for Gaussian channels. Consequently, we must rewrite our multimodal model in a way that is suitable for parameter estimation.

In our models (1) and (3) the temporal responses *r*(*t*) are not in a parametric form. We can address this by parameterizing them as a weighted sum of *B* basis functions:

\begin{align*} r\left(t\right) = \sum_{b = 1}^B w_b f_b\left(t\right) \, . \end{align*}

In this way, the temporal response is parameterized by the weights $w_1, \ldots, w_B$. In this work, the basis functions $f_b(t)$ are 10 truncated Gaussians with a standard deviation of 100 ms, with means evenly spread out from −500 ms to 500 ms relative to the event time *t_s_*.

Now that our model has been parameterized, we need a way to represent the independent variables, the event classes *s* and times *t_s_*, in a way that is suitable for generalized linear regression. To do this, we define a signal $d_n(t)$ that encodes both the event classes and the event times:

\begin{align*} d_n\left(t\right) = \sum_{t_s \in \tau} \delta\left(t - t_s\right) s_n\left(t_s\right). \end{align*}

Here, $s_n(t)$ is the *n*th element of the one-hot vector *s* at time *t* and *τ* is the set of event times. Using the parameterization of *r*(*t*) in (A.1) and the event-encoding signal *d*(*t*) in (A.2), the terms $\big(\sum_{t_s \in T}r(t-t_s)\big)\phi^Ts(t)$ in (5) can be rewritten as a matrix multiplication, in a manner similar to [60]:

\begin{align*} \begin{split} &amp;\left(\sum_{t_s \in \tau}r\left(t-t_s\right)\right)\phi^Ts\left(t\right) = \left(\sum_{t_s \in \tau}r\left(t-t_s\right)\right)s^T\left(t\right)\phi \\ &amp;\quad = \left[ \left(\sum_{t_s \in \tau}r\left(t-t_s\right)\right)s_1\left(t\right)\, , \, \ldots \, ,\, \left(\sum_{t_s \in \tau}r\left(t-t_s\right)\right)s_n\left(t\right) \right]\phi \\ &amp;\quad = \left[r\left(t\right) * d_1\left(t\right) \, , \, \ldots \, , \, r\left(t\right) * d_n\left(t\right) \right] \phi \\ &amp;\quad = \left[\sum_{i = 1}^b w_i f_i\left(t\right) * d_1\left(t\right) \, , \, \ldots \, , \, \sum_{i = 1}^b w_i f_i\left(t\right) * d_n\left(t\right) \right] \phi \\ &amp;\quad = \left[w_1 \, \ldots \, w_n\right] \begin{bmatrix} f_1 * d_1\left(t\right) &amp; \ldots &amp; f_1 * d_n\left(t\right) \\ \vdots &amp; \ddots &amp; \vdots \\ f_b * d_1\left(t\right) &amp; \ldots &amp; f_b * d_n\left(t\right) \end{bmatrix} \phi \\ &amp;\quad = \mathbf{w}^T X(t) \phi \, . \end{split} \end{align*}

Here, $*$ is the convolution operation and $\mathbf{w} = [w_1 \ldots w_b]^T$ is a vector of the temporal response parameters. The matrix *X*(*t*) encodes the independent variables *t_s_* and *s*, which will be known for training data, along with our chosen basis functions $f_b(t)$. Substituting this into (1) and (3), we obtain a generalized bilinear model for point process channels:

\begin{align*} \log \lambda_i\left(t\right) = \mathbf{w}_i^T X\left(t\right) \phi_i + \alpha_i \end{align*}

and a bilinear model for Gaussian channels:

\begin{align*} y_j\left(t\right) = \mathbf{w}_j^T X\left(t\right) \phi_j + w_j\left(t\right) \, , \, w_j\left(t\right) \sim \mathcal{N}\left(0, \sigma_j^2\right) \end{align*}

**w** and *φ* are parameter vectors that represent the temporal and spatial responses respectively, and must be estimated from the training dataset. Generalized bilinear models have previously been used to model the spatiotemporal responses of spiking neurons [60, 98].

If we fix the value of *w*, then we can learn *φ* by fitting a linear model or Poisson GLM with $\mathbf{w}_i^T X(t)$ as the independent variable and the training data, either Gaussian or point-process, as the dependent variable. We can similarly learn *w* by fixing *φ* and using $X(t) \phi_i$ as the independent variable. In this work, we fit parameters by iteratively fixing either the spatial or temporal response and fitting the other, as in prior works [60, 98]. Parameters were initialized to vectors of all ones.

For point-process signals, *α* is learned as the bias parameter in the GLM, and for Gaussian signals, *σ* is the standard deviation of the residuals after the parameters are learned.

## Appendix B Noise sensitivity analysis

We repeated the simulation analysis presented in section 3.1 for varying levels of spike and LFP noise in order to investigate how MED performance is affected. We varied the LFP noise level while keeping the spike noise level fixed, and then varied the spike noise level while keeping the LFP noise level fixed. We adjusted the LFP noise level by varying the noise variance parameter *σ* from 20 to 80 while fixing the maximum signal amplitude at 1 (arbitrary units). Similarly, we adjusted the spiking noise level by varying the baseline firing rate from 40 Hz to 70 Hz while fixing the maximum signal firing rate at 100 Hz. The results of this analysis are shown in figure B1. We found that while unimodal performance declines as the corresponding noise levels are increased, the MED is still able to combine information from both modalities and increase performance as channels of either type are added.
